# Manual curation for improved genome annotation of the functionally extinct northern white rhinoceros (*Ceratotherium simum cottoni*)

**DOI:** 10.1371/journal.pone.0340594

**Published:** 2026-01-05

**Authors:** Elena Ruggeri, Julien Prunier, Marc-André Sirard, Barbara Durrant, Kristin Klohonatz

**Affiliations:** 1 Reproductive Sciences, Conservation Science Wildlife Health, San Diego Zoo Wildlife Alliance, Escondido, California, United States of America; 2 Genovalia, Centre de valorisation des données en génomique non-humaine, Université Laval, Québec City, Québec, Canada; 3 Département des Sciences Animales, Université Laval, Québec City, Québec, Canada; 4 Center for Research on Reproduction and Women’s Health, Perelman School of Medicine, University of Pennsylvania, Philadelphia, Pennsylvania, United States of America; Justus Liebig Universitat Giessen, GERMANY

## Abstract

The northern white rhinoceros (*Ceratotherium simum cottoni*) genome and annotation were previously published, but the annotation contained few genes, with many annotation misalignments, and nomenclature not matching HGNC/VGNC naming conventions, making transcriptional studies very difficult. We used *in vivo* collected granulosa cells for RNA sequencing and de novo transcript assembly through StringTie to identify all nucleotide gene sequences in our samples. Through extensive manual curation we were able to generate a greatly improved genome annotation, increasing gene numbers by 81%. This will greatly enable researchers in this field to utilize the genome and annotation to complete transcriptional studies with this species.

## Background

White rhinoceroses play a key ecological role within African ecosystems. An enduring conservation plan has been unraveled to reverse the vanishing of this keystone species [1–8]. Poaching remains the primary threat, with the northern white rhinoceros (*Ceratotherium simum cottoni*, NWR) population functionally extinct, and the southern white rhinoceros (*Ceratotherium simum simum,* SWR) threatened. Over the past decade, significant conservation efforts have focused on saving the critically endangered NWR culminating in the successful application of assisted reproductive technologies (ARTs), including the in vitro production of blastocysts from the final two surviving females [1,2]. These advancements were made possible largely due to the SWR, a closely related subspecies that has served as a critical model in developing these techniques [2]. Although the NWR and SWR have been geographically isolated for thousands of years, recent studies confirm that their genomes are remarkably similar at the chromosomal and genomic level [9]. This genetic compatibility makes the SWR a promising candidate for developing and applying reproductive strategies aimed at rescuing the NWR. Genomic resources, such as the newly published NWR genome [9], transcriptomic studies on reproductive cells from SWR [5,7,8], and the ongoing development of induced pluripotent stem cells (iPSCs) [9], are available to contribute to conservation efforts for rhinoceroses.

Genomic strategies and assisted reproductive technologies are essential to counteract loss of biodiversity, hence the urgency in continuing to develop and advance conservation strategies for this species. The availability of the NWR reference genome marked a milestone in wildlife genomics and was published in early 2025 [10], allowing white rhinoceros research to further integrate molecular genetic approaches. This study highlighted the close genetic homology between the NWR and SWR [10], leading to the interchangeable use of their genomes for research purposes. The recently published genome and annotation were limited due to sample generation and computational pipeline. The annotation was not thorough and contained various gene nomenclatures, which led to large difficulties in developing and interpreting transcriptional studies. Furthermore, this annotation was generated using BRAKER3 [11] and protein coding sequences were generated from human, mouse, southern white rhinoceros, domestic horse, and blue whale proteins. Of the 14,274 annotated transcripts, only 7,299 (51%) were called to the correct genetic sequence; 6,763 were assigned to protein names (rendering them difficult to use in downstream pathway analysis); 212 were incorrectly assigned to sequences; and an additional 455 transcripts were assigned to bacterial genes. Due to these faults, an urgent need to improve the annotation arose.

To improve the NWR genome annotation, we sequenced RNA from granulosa cells, which are reproductive cells, at a variety of developmental stages, promising a larger diversity of transcripts to be sequenced compared to the previous annotation [10]. Sequencing reads were assembled into transcripts aligned to the NWR genome. This analysis of transcriptionally active cells allowed the identification of a significant number of genes that led to a more thorough annotation, allowing future genomic studies to advance the ongoing conservation work in this species [5,7,8].

## Results and discussion

Total RNA was extracted and sequenced using a paired-end short read technology. The sequencing depth for each sample averaged 37,364,052 (Table 1). Those reads were then aligned to the northern white rhinoceros (NWR) genome and assembled into transcripts which were manually curated based on homology with other rhinoceros species (*Ceratotherium simum simum* and *Diceros bicornis*) and the phylogenetically related *Equus Caballus.*

**Table 1:** The sequencing depth of samples used for annotation generation.

GSE Reference	Sequencing Depth
GSE261038	36,411,992
GSE261038	51,216,699
GSE261038	46,272,140
GSE261038	35,669,810
GSE261038	40,559,472
GSE261038	40,645,864
GSE261038	34,809,961
GSE261038	63,608,715
GSE300824	30,218,485
GSE300824	26,007,749
GSE300824	36,185,295
GSE300824	33,485,901
GSE300824	24,585,400
GSE300824	23,419,242

**Fig 1:**
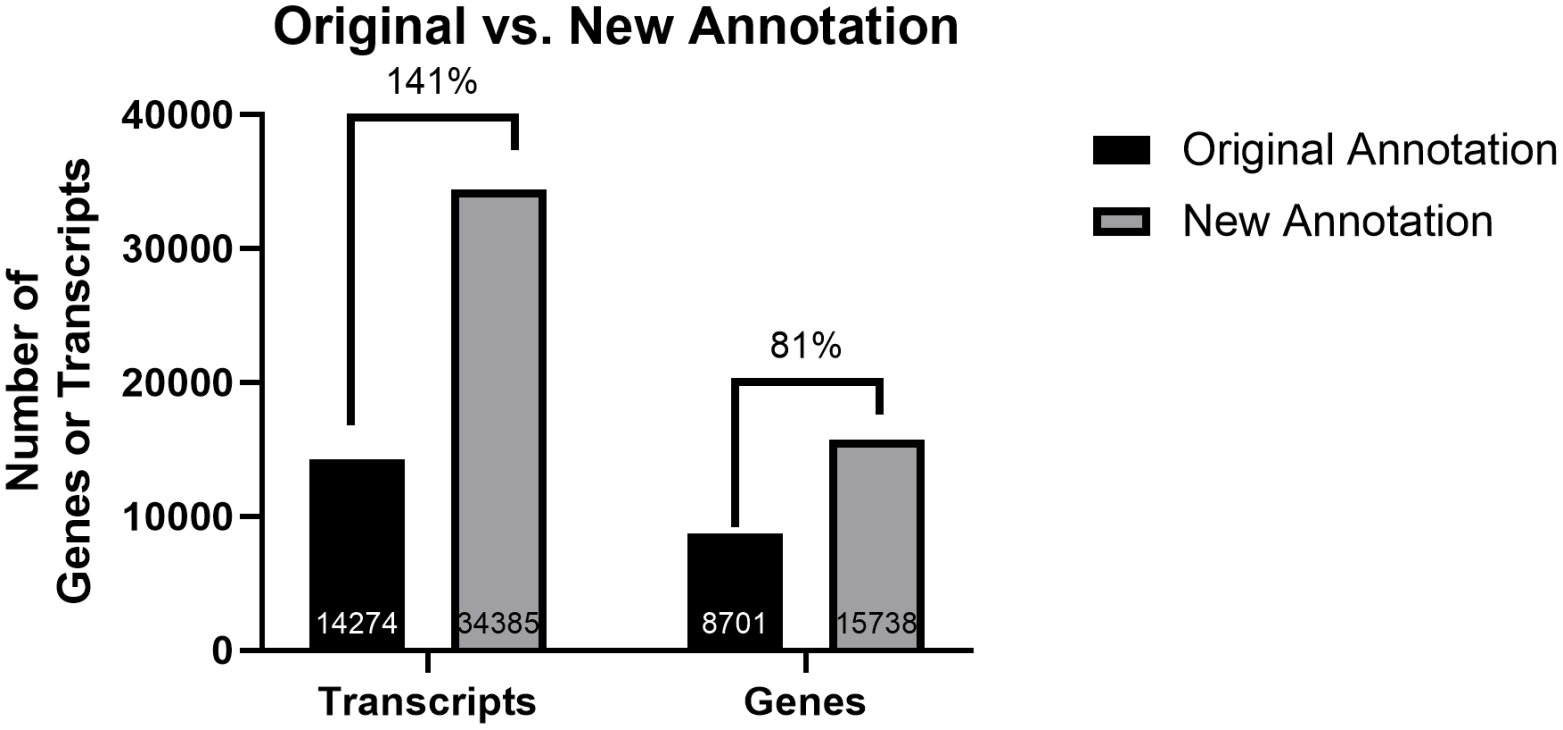
Improvement of the northern white rhinoceros genome annotation before and after manual curation. The values represent the percent increase from the original to the new annotation.

As expected, given the sampling of granulosa cells at various developmental stages, the final annotation after manual curation resulted in a large increase in both annotated transcripts and genes. The original annotation contained 14,274 functional transcripts, while the new annotation contains 34,385 functional transcripts, an increase of 141% (Table 2). Following the same trend, the original annotation contained only 8,701 functional genes, whereas after manual curation the gene number increased by 81% resulting in 15,738 functional genes in the new annotation (Fig 1). This substantial increase was primarily due to many annotated genes that were not included in the first annotation but were detected as “de novo” transcripts in the granulosa cells. While the total number of identified genes increased substantially, reaching a number closer to other well-known mammalian genomes (e.g., 20,848 gene models in cow, Ensembl release 113), the average transcript length remained unchanged (Table 2), supporting the quality of the bioinformatic work. The total sequence length for all transcripts represented 48% of the genome assembly which may appear high but is well aligned with other reported transcriptome lengths such as in rat or human, for instance [12,13]. This is likely related to our total RNA extraction and sequencing that resulted in a transcriptome including unspliced transcripts. In addition, this transcriptome included some repeated elements (including found LINES) and we cannot entirely discard the possibility of pseudogenes (full-length or truncated sequences that may be transcribed but not translated into functional coding sequences). Altogether, we significantly improved the genome annotation through the identification of many additional gene sequences.

**Table 2:** Descriptive Parameters of the Northern White Rhinoceros Genome Annotation.

	New Annotation	Original Annotation
Annotated Genes	15,738	8,701
Number of Transcripts	34,385	14,274
Average Transcript Length	36,981 bp	39,878 bp
Percent of Genome Covered by Exons	48%	1%

After evaluating the sequences of the “de novo” transcriptome, many sequences originated from specific genes and assigned appropriately. Gene names were also corrected from the previous annotation to reflect HGNC/VGNC (HUGO gene nomenclature committee/vertebrate gene nomenclature) naming conventions. BUSCO analysis revealed a completeness of 93.4% (single copy 89.4%, duplicated 3.9%), supporting the quality and completeness of our transcriptome. This resulted in an annotation that is more operational and complete for transcriptional studies in this species.

## Conclusions

We significantly improved the annotation of the northern white rhinoceros genome using RNAseq mapping and transcriptome assembly. This new annotation of the northern white rhinoceros genome CerSimCot1.0 (GCA_021442165.1) is now available and will undoubtfully aid genomic studies and conservation strategies for this and closely related species.

## Methods

Four southern white rhinoceros females were anesthetized, and transrectal ovum pickup (OPU) was performed as previously described [5–7]. All procedures, experiments, and methods were reviewed and approved by San Diego Zoo Wildlife Alliance’s Institutional Animal Care and Use Committee (IACUC; protocol number 18−018). To alleviate any pain, all animals underwent anesthesia protocols that were described in the previously referenced publication [7]. OPU was achieved using a customized, ultrasound-guided probe fitted with double-lumen needles. The follicles were first ablated, then the contents were aspirated and rinsed with a warm (37°C) flushing solution (Vigro) containing 12.5 I.U./mL of heparin. After OPU and the oocytes were isolated from the collection, fluid and free-floating mural granulosa cells were collected and pipetted directly into RNAlater (Thermo Fisher Scientific, Waltham, MA). In total, granulosa cells from ten follicles were utilized for this study and a total of 14 tubes (samples) were used for RNA isolation and sequencing. In more detail, 4 rhino samples (NCBI Bioproject: GSE261038) were run in technical replicates with RNA being isolated from separately stored pools of granulosa cells totaling up to 8 granulosa cell samples. From GSE300824 the samples were not run in technical replicates, resulting in a total of six granulosa cell samples. Over the two experiments, we obtained two growing, six dominant, and two pre-ovulatory follicles represented. Total RNA was isolated from granulosa cells using an Arcturus PicoPure RNA Isolation Kit (Thermo Fisher Scientific, Waltham, MA) per the manufacturer’s instructions. A Qubit 4 Fluorometer (Thermo Fischer Scientific, Waltham, MA) was used for quantification, and a 4150 TapeStation System (Agilent Technologies, Santa Clara, CA) was used to determine RNA integrity number (RIN) values. Only samples with a RIN greater than 6.0 were used for RNA-Seq analysis. Library preparation and RNA sequencing were performed at the University of California San Diego Institute for Genomic Medicine Center. Following the manufacturer’s protocol, RNA-sequencing (cDNA) libraries were prepared using the Illumina TruSeq Stranded Total RNA Prep with Ribo-Zero Plus for six samples. The libraries were sequenced as 100 bp paired-end reads on an Illumina NovaSeq 6000 (Illumina, San Diego, CA). The raw data files were uploaded to the Gene Expression Omnibus under accession number GSE261038. The remaining eight libraries were prepared per the manufacturer’s protocol using the NovaSeq X plus, Illumina stranded Total RNA with Ribozero plus library preparation kit. These prepared libraries were sequenced as 150 bp paired-end reads on an Illumina NovaSeq 6000 (Illumina, San Diego, CA). The raw data files were uploaded to the Gene Expression Omnibus under accession number GSE300824. Bioinformatic analysis was performed on the Galaxy web platform using the public server usegalaxy.org [14]. HISAT2 was used to align reads to the northern white rhinoceros (NWR) genome CerSimCot1.0 (GCA_021442165.1) [10,15,16]. Transcript assembly for both annotated and unannotated transcripts was performed with Stringtie2, and each animal sample was analyzed individually [17]. The resulting transcripts for individual samples were merged to create a final annotation file representing the union of all samples [17]. The generated annotation file was used for subsequent manual curation. The complete Galaxy workflow can be found in Supplemental Material 1. Manual curation was performed by determining the nucleotide sequence for each region identified with the merged annotation file in StringTie [17] without a gene assigned. These sequences were searched individually in NCBI BLAST [18] for homology between other rhinoceros species (*Ceratotherium simum simum* and *Diceros bicornis*) and the phylogenetically related *Equus Caballus* [10]. If a sequence had a percent identity 80% [19], or greater, to a known gene, the sequence was properly assigned to that gene. In addition, regions that were assigned to a gene from Wang et. al [20] were reevaluated through the same manual curation process. Erroneously assigned genes, genes with protein nomenclature (rendering it unusable for pathway analyses), and bacterial genes were discovered during manual curation and these errors were corrected. Genes were named using the HGNC/VGNC naming conventions for ease of use for further transcriptional studies. Transcriptome completion was assessed using a BUSCO analysis [21] analysis. The resulting annotation was made publicly available in the following GitHub repository (https://github.com/eruggeri/Northern-White-Rhinoceros-Annotation).

## Supporting information

S1 File Galaxy workflow used to generate annotated and unannotated sequences. This file contains the code lines for the workflow used in Galaxy to generate the annotated and unannotated sequences for the annotation file.
(PDF)
