# Supplementary material for: Manual curation for improved genome annotation of the functionally extinct northern white rhinoceros (Ceratotherium simum cottoni)
Source: PLoS One. 2026 Jan 5;21(1):e0340594. doi: 10.1371/journal.pone.0340594 (PMC12768360; doi:10.1371/journal.pone.0340594)
Supplement: S1 File — This file contains the code lines for the workflow used in Galaxy to generate the annotated and unannotated sequences for the annotation file. (PDF) [file pone.0340594.s001.pdf]

Supplementary Material 1. Galaxy workflow used to generate annotated and unannotated sequences.

{
  "a_galaxy_workflow": "true",
  "annotation": "",
  "comments": [],
  "format-version": "0.1",
  "name": "NWR Genome Annotation",
  "report": {
    "markdown": "\n# Workflow Execution Report\n\n## Workflow Inputs\n```galaxy\ninvocation_inputs()\n```\n\n## Workflow Outputs\n```galaxy\ninvocation_outputs()\n```\n\n## Workflow\n```galaxy\nworkflow_display()\n```\n"
  },
  "steps": {
    "0": {
      "annotation": "",
      "content_id": "toolshed.g2.bx.psu.edu/repos/devteam/fastqc/fastqc/0.74+galaxy1",
      "errors": null,
      "id": 0,
      "input_connections": {},
      "inputs": [
        {
          "description": "runtime parameter for tool FastQC",
          "name": "adapters"
        },
        {
          "description": "runtime parameter for tool FastQC",
          "name": "contaminants"
        },
        {
          "description": "runtime parameter for tool FastQC",
          "name": "input_file"
        },
        {
          "description": "runtime parameter for tool FastQC",
          "name": "limits"
        }
      ],
      "label": null,
      "name": "FastQC",
      "outputs": [
        {

"name": "html_file",
"type": "html"
},
{
"name": "text_file",
"type": "txt"
}
],
"position": {
"left": 0.0,
"top": 0.0
},
"post_job_actions": {},
"tool_id": "toolshed.g2.bx.psu.edu/repos/devteam/fastqc/fastqc/0.74+galaxy1",
"tool_shed_repository": {
"changeset_revision": "2c64fded1286",
"name": "fastqc",
"owner": "devteam",
"tool_shed": "toolshed.g2.bx.psu.edu"
},
"tool_state": "{\"adapters\": {\"__class__\": \"RuntimeValue\"}, \"contaminants\": {\"__class__\": \"RuntimeValue\"}, \"input_file\": {\"__class__\": \"RuntimeValue\"}, \"kmers\": \"7\", \"limits\": {\"__class__\": \"RuntimeValue\"}, \"min_length\": null, \"nogroup\": false, \"__page__\": 0, \"__rerun_remap_job_id__\": null}",
"tool_uuid": null,
"tool_version": "0.74+galaxy1",
"type": "tool",
"uuid": "6de97f10-36ec-4c15-8d4e-12dc97d9b886",
"when": null,
"workflow_outputs": []
},
"1": {
"annotation": "",
"content_id": "toolshed.g2.bx.psu.edu/repos/iuc/multiqc/multiqc/1.27+galaxy4",
"errors": null,
"id": 1,
"input_connections": {},
"inputs": [
{
"description": "runtime parameter for tool MultiQC",
"name": "image_content_input"
}
],
"label": null,

"name": "MultiQC",
"outputs": [
  {
    "name": "html_report",
    "type": "html"
  },
  {
    "name": "stats",
    "type": "tabular"
  }
],
"position": {
  "left": 232.22574869791663,
  "top": 0.024312337239564386
},
"post_job_actions": {},
"tool_id": "toolshed.g2.bx.psu.edu/repos/iuc/multiqc/multiqc/1.27+galaxy4",
"tool_shed_repository": {
  "changeset_revision": "e4dd4c0622f6",
  "name": "multiqc",
  "owner": "iuc",
  "tool_shed": "toolshed.g2.bx.psu.edu"
},
"tool_state": "{\"comment\": \"\", \"export\": false, \"flat\": false,
\"image_content_input\": {\"__class__\": \"RuntimeValue\"}, \"png_plots\": false, \"results\":
[{\"__index__\": 0, \"software_cond\": {\"software\": \"bamtools\", \"__current_case__\": 0,
\"input\": {\"__class__\": \"RuntimeValue\"}}}], \"title\": \"\", \"__page__\": 0,
\"__rerun_remap_job_id__\": null}",
"tool_uuid": null,
"tool_version": "1.27+galaxy4",
"type": "tool",
"uuid": "30e7ac35-7746-4c6a-ae7c-0749cf235a81",
"when": null,
"workflow_outputs": []
},
"2": {
  "annotation": "",
  "content_id":
"toolshed.g2.bx.psu.edu/repos/pjbriggs/trimmomatic/trimmomatic/0.39+galaxy2",
  "errors": null,
  "id": 2,
  "input_connections": {},
  "inputs": [
    {

"description": "runtime parameter for tool Trimmomatic",
"name": "readtype"
},
{
"description": "runtime parameter for tool Trimmomatic",
"name": "readtype"
}
],
"label": null,
"name": "Trimmomatic",
"outputs": [
{
"name": "fastq_out_r1_paired",
"type": "input"
},
{
"name": "fastq_out_r2_paired",
"type": "input"
},
{
"name": "fastq_out_r1_unpaired",
"type": "input"
},
{
"name": "fastq_out_r2_unpaired",
"type": "input"
}
],
"position": {
"left": 465.55209689670136,
"top": 1.1146036783854356
},
"post_job_actions": {},
"tool_id":
"toolshed.g2.bx.psu.edu/repos/pjbriggs/trimmomatic/trimmomatic/0.39+galaxy2",
"tool_shed_repository": {
"changeset_revision": "b9aaed85cbd1",
"name": "trimmomatic",
"owner": "pjbriggs",
"tool_shed": "toolshed.g2.bx.psu.edu"
},
"tool_state": "{\"illuminaclip\": {\"do_illuminaclip\": \"yes\", \"__current_case__\": 0,
\"adapter_type\": {\"standard_or_custom\": \"standard\", \"__current_case__\": 0,
\"adapter_fasta\": \"NexteraPE-PE.fa\"}, \"seed_mismatches\": \"2\",

\"palindrome_clip_threshold\": \"30\", \"simple_clip_threshold\": \"10\", \"min_adapter_len\": \"8\", \"keep_both_reads\": true}, \"operations\": [{\"__index__\": 0, \"operation\": {\"name\": \"CROP\", \"__current_case__\": 4, \"crop\": \"140\"}}, {\"__index__\": 1, \"operation\": {\"name\": \"HEADCROP\", \"__current_case__\": 5, \"headcrop\": \"10\"}}, {\"__index__\": 2, \"operation\": {\"name\": \"MINLEN\", \"__current_case__\": 1, \"minlen\": \"20\"}}], \"output_err\": false, \"output_logs\": false, \"quality_score\": null, \"readtype\": {\"single_or_paired\": \"pair_of_files\", \"__current_case__\": 1, \"fastq_r1_in\": {\"__class__\": \"RuntimeValue\"}, \"fastq_r2_in\": {\"__class__\": \"RuntimeValue\"}}, \"__page__\": 0, \"__rerun_remap_job_id__\": null}",
      "tool_uuid": null,
      "tool_version": "0.39+galaxy2",
      "type": "tool",
      "uuid": "6fd8dd3c-cf86-4bcc-9269-36f2b758bc6d",
      "when": null,
      "workflow_outputs": []
    },
    "3": {
      "annotation": "",
      "content_id": "toolshed.g2.bx.psu.edu/repos/devteam/fastqc/fastqc/0.74+galaxy1",
      "errors": null,
      "id": 3,
      "input_connections": {},
      "inputs": [
        {
          "description": "runtime parameter for tool FastQC",
          "name": "adapters"
        },
        {
          "description": "runtime parameter for tool FastQC",
          "name": "contaminants"
        },
        {
          "description": "runtime parameter for tool FastQC",
          "name": "input_file"
        },
        {
          "description": "runtime parameter for tool FastQC",
          "name": "limits"
        }
      ],
      "label": null,
      "name": "FastQC",
      "outputs": [
        {

"name": "html_file",
"type": "html"
},
{
"name": "text_file",
"type": "txt"
}
],
"position": {
"left": 685.546875,
"top": 3.3420477973090215
},
"post_job_actions": {},
"tool_id": "toolshed.g2.bx.psu.edu/repos/devteam/fastqc/fastqc/0.74+galaxy1",
"tool_shed_repository": {
"changeset_revision": "2c64fded1286",
"name": "fastqc",
"owner": "devteam",
"tool_shed": "toolshed.g2.bx.psu.edu"
},
"tool_state": "{\"adapters\": {\"__class__\": \"RuntimeValue\"}, \"contaminants\": {\"__class__\": \"RuntimeValue\"}, \"input_file\": {\"__class__\": \"RuntimeValue\"}, \"kmers\": \"7\", \"limits\": {\"__class__\": \"RuntimeValue\"}, \"min_length\": null, \"nogroup\": false, \"__page__\": 0, \"__rerun_remap_job_id__\": null}",
"tool_uuid": null,
"tool_version": "0.74+galaxy1",
"type": "tool",
"uuid": "74d24d14-8404-4242-a055-cfd323126ee9",
"when": null,
"workflow_outputs": []
},
"4": {
"annotation": "",
"content_id": "toolshed.g2.bx.psu.edu/repos/iuc/multiqc/multiqc/1.27+galaxy4",
"errors": null,
"id": 4,
"input_connections": {},
"inputs": [
{
"description": "runtime parameter for tool MultiQC",
"name": "image_content_input"
}
],
"label": null,

"name": "MultiQC",
"outputs": [
{
"name": "html_report",
"type": "html"
},
{
"name": "stats",
"type": "tabular"
}
],
"position": {
"left": 910.2070792411841,
"top": 2.7241475352905127
},
"post_job_actions": {},
"tool_id": "toolshed.g2.bx.psu.edu/repos/iuc/multiqc/multiqc/1.27+galaxy4",
"tool_shed_repository": {
"changeset_revision": "e4dd4c0622f6",
"name": "multiqc",
"owner": "iuc",
"tool_shed": "toolshed.g2.bx.psu.edu"
},
"tool_state": "{\"comment\": \"\", \"export\": false, \"flat\": false,
\"image_content_input\": {\"__class__\": \"RuntimeValue\"}, \"png_plots\": false, \"results\":
[{\"__index__\": 0, \"software_cond\": {\"software\": \"bamtools\", \"__current_case__\": 0,
\"input\": {\"__class__\": \"RuntimeValue\"}}}], \"title\": \"\", \"__page__\": 0,
\"__rerun_remap_job_id__\": null}",
"tool_uuid": null,
"tool_version": "1.27+galaxy4",
"type": "tool",
"uuid": "761f7867-273b-4b83-825f-cd7a4ba858fb",
"when": null,
"workflow_outputs": []
},
"5": {
"annotation": "",
"content_id": "toolshed.g2.bx.psu.edu/repos/iuc/hisat2/hisat2/2.2.1+galaxy1",
"errors": null,
"id": 5,
"input_connections": {},
"inputs": [
{
"description": "runtime parameter for tool HISAT2",

```
            "name": "library"
          },
          {
            "description": "runtime parameter for tool HISAT2",
            "name": "library"
          }
        ],
        "label": null,
        "name": "HISAT2",
        "outputs": [
          {
            "name": "output_alignments",
            "type": "bam"
          }
        ],
        "position": {
          "left": 1132.0190016860008,
          "top": 4.037201205045164
        },
        "post_job_actions": {},
        "tool_id": "toolshed.g2.bx.psu.edu/repos/iuc/hisat2/hisat2/2.2.1+galaxy1",
        "tool_shed_repository": {
          "changeset_revision": "f4af63aaf57a",
          "name": "hisat2",
          "owner": "iuc",
          "tool_shed": "toolshed.g2.bx.psu.edu"
        },
        "tool_state": "{\"adv\": {\"input_options\": {\"input_options_selector\": \"defaults\", \"__current_case__\": 0}, \"alignment_options\": {\"alignment_options_selector\": \"defaults\", \"__current_case__\": 0}, \"scoring_options\": {\"scoring_options_selector\": \"defaults\", \"__current_case__\": 0}, \"spliced_options\": {\"spliced_options_selector\": \"defaults\", \"__current_case__\": 0}, \"reporting_options\": {\"reporting_options_selector\": \"defaults\", \"__current_case__\": 0}, \"output_options\": {\"output_options_selector\": \"defaults\", \"__current_case__\": 0}, \"sam_options\": {\"sam_options_selector\": \"defaults\", \"__current_case__\": 0}, \"other_options\": {\"other_options_selector\": \"defaults\", \"__current_case__\": 0}}, \"library\": {\"type\": \"paired\", \"__current_case__\": 1, \"input_1\": {\"__class__\": \"RuntimeValue\"}, \"input_2\": {\"__class__\": \"RuntimeValue\"}, \"rna_strandness\": \"\", \"paired_options\": {\"paired_options_selector\": \"defaults\", \"__current_case__\": 0}}, \"reference_genome\": {\"source\": \"indexed\", \"__current_case__\": 0, \"index\": \"apiMel4\"}, \"sum\": {\"new_summary\": false, \"summary_file\": false}, \"__page__\": 0, \"__rerun_remap_job_id__\": null}",
        "tool_uuid": null,
        "tool_version": "2.2.1+galaxy1",
```

"type": "tool",
"uuid": "602861a1-30cc-45ad-bc0a-d9b78d3df6d5",
"when": null,
"workflow_outputs": []
},
"6": {
"annotation": "",
"content_id": "toolshed.g2.bx.psu.edu/repos/iuc/stringtie/stringtie/2.2.3+galaxy0",
"errors": null,
"id": 6,
"input_connections": {},
"inputs": [
{
"description": "runtime parameter for tool StringTie",
"name": "adv"
},
{
"description": "runtime parameter for tool StringTie",
"name": "input_options"
}
],
"label": null,
"name": "StringTie",
"outputs": [
{
"name": "output_gtf",
"type": "gtf"
}
],
"position": {
"left": 1350.661445191369,
"top": 2.734945407959742
},
"post_job_actions": {},
"tool_id": "toolshed.g2.bx.psu.edu/repos/iuc/stringtie/stringtie/2.2.3+galaxy0",
"tool_shed_repository": {
"changeset_revision": "cbf488da3b2c",
"name": "stringtie",
"owner": "iuc",
"tool_shed": "toolshed.g2.bx.psu.edu"
},
"tool_state": "{\"adv\": {\"abundance_estimation\": false, \"omit_sequences\": \"\",
\"name_prefix\": null, \"fraction\": \"0.01\", \"min_tlen\": \"200\", \"min_anchor_len\": \"10\",
\"min_anchor_cov\": \"1\", \"min_bundle_cov\": \"1\", \"bdist\": \"50\", \"bundle_fraction\":

\"1.0\", \"disable_trimming\": false, \"multi_mapping\": false, \"point_features\": {\"__class__\": \"RuntimeValue\"}}, \"guide\": {\"use_guide\": \"yes\", \"__current_case__\": 1, \"guide_source\": {\"guide_gff_select\": \"history\", \"__current_case__\": 1, \"ref_hist\": {\"__class__\": \"RuntimeValue\"}}, \"input_estimation\": false, \"special_outputs\": {\"special_outputs_select\": \"no\", \"__current_case__\": 2}, \"coverage_file\": false}, \"input_options\": {\"input_mode\": \"short_reads\", \"__current_case__\": 0, \"input_bam\": {\"__class__\": \"RuntimeValue\"}}, \"rna_strandness\": \"\", \"__page__\": 0, \"__rerun_remap_job_id__\": null}",
"tool_uuid": null,
"tool_version": "2.2.3+galaxy0",
"type": "tool",
"uuid": "dfabf2c6-62c0-44d8-ae11-c273069e2f5f",
"when": null,
"workflow_outputs": []
},
"7": {
"annotation": "",
"content_id":
"toolshed.g2.bx.psu.edu/repos/iuc/stringtie/stringtie_merge/2.2.3+galaxy0",
"errors": null,
"id": 7,
"input_connections": {},
"inputs": [
{
"description": "runtime parameter for tool StringTie merge",
"name": "guide_gff"
},
{
"description": "runtime parameter for tool StringTie merge",
"name": "input_gtf"
}
],
"label": null,
"name": "StringTie merge",
"outputs": [
{
"name": "out_gtf",
"type": "gtf"
}
],
"position": {
"left": 1581.2335563641714,
"top": 4.064013900100235
},

"post_job_actions": {},
"tool_id":
"toolshed.g2.bx.psu.edu/repos/iuc/stringtie/stringtie_merge/2.2.3+galaxy0",
"tool_shed_repository": {
"changeset_revision": "cbf488da3b2c",
"name": "stringtie",
"owner": "iuc",
"tool_shed": "toolshed.g2.bx.psu.edu"
},
"tool_state": "{\"gap_len\": \"250\", \"guide_gff\": {\"__class__\": \"RuntimeValue\"},
\"input_gtf\": {\"__class__\": \"RuntimeValue\"}, \"keep_introns\": false, \"min_cov\": \"0\",
\"min_fpkm\": \"1.0\", \"min_iso\": \"0.01\", \"min_len\": \"50\", \"min_tpm\": \"1.0\",
\"__page__\": 0, \"__rerun_remap_job_id__\": null}",
"tool_uuid": null,
"tool_version": "2.2.3+galaxy0",
"type": "tool",
"uuid": "1c7d4bb2-88bd-44a3-94fc-5794768081bf",
"when": null,
"workflow_outputs": []
},
"8": {
"annotation": "",
"content_id": "toolshed.g2.bx.psu.edu/repos/iuc/stringtie/stringtie/2.2.3+galaxy0",
"errors": null,
"id": 8,
"input_connections": {},
"inputs": [
{
"description": "runtime parameter for tool StringTie",
"name": "adv"
},
{
"description": "runtime parameter for tool StringTie",
"name": "input_options"
}
],
"label": null,
"name": "StringTie",
"outputs": [
{
"name": "output_gtf",
"type": "gtf"
},
{

"name": "gene_counts",
"type": "tabular"
},
{
"name": "transcript_counts",
"type": "tabular"
}
],
"position": {
"left": 1798.565534453908,
"top": 2.71686807056966
},
"post_job_actions": {},
"tool_id": "toolshed.g2.bx.psu.edu/repos/iuc/stringtie/stringtie/2.2.3+galaxy0",
"tool_shed_repository": {
"changeset_revision": "cbf488da3b2c",
"name": "stringtie",
"owner": "iuc",
"tool_shed": "toolshed.g2.bx.psu.edu"
},
"tool_state": "{\"adv\": {\"abundance_estimation\": false, \"omit_sequences\": \"\", \"name_prefix\": null, \"fraction\": \"0.01\", \"min_tlen\": \"200\", \"min_anchor_len\": \"10\", \"min_anchor_cov\": \"1\", \"min_bundle_cov\": \"1\", \"bdist\": \"50\", \"bundle_fraction\": \"1.0\", \"disable_trimming\": false, \"multi_mapping\": false, \"point_features\": {\"__class__\": \"RuntimeValue\"}}, \"guide\": {\"use_guide\": \"yes\", \"__current_case__\": 1, \"guide_source\": {\"guide_gff_select\": \"history\", \"__current_case__\": 1, \"ref_hist\": {\"__class__\": \"RuntimeValue\"}}, \"input_estimation\": true, \"special_outputs\": {\"special_outputs_select\": \"deseq2\", \"__current_case__\": 1, \"read_length\": \"75\", \"clustering\": false, \"string\": null, \"key\": null}, \"coverage_file\": false}, \"input_options\": {\"input_mode\": \"short_reads\", \"__current_case__\": 0, \"input_bam\": {\"__class__\": \"RuntimeValue\"}}, \"rna_strandness\": \"\", \"__page__\": 0, \"__rerun_remap_job_id__\": null}",
"tool_uuid": null,
"tool_version": "2.2.3+galaxy0",
"type": "tool",
"uuid": "7f0d8810-dbef-4bab-a718-6c0effa81e8a",
"when": null,
"workflow_outputs": []
}
},
"tags": [],
"uuid": "b7231de5-1717-4430-8f76-f11665d7834e",
"version": 0
}
